# Effect of uncultured adipose-derived stromal vascular fraction on preventing urethral stricture formation in rats

**DOI:** 10.1038/s41598-022-07472-1

**Published:** 2022-03-04

**Authors:** Liuhua Zhou, Tianli Yang, Feng Zhao, Kaiwei Song, Luwei Xu, Zhongle Xu, Changcheng Zhou, Zhiqiang Qin, Zheng Xu, Ran Wu, Hua Xu, Ruipeng Jia

**Affiliations:** 1 Department of Urology, Nanjing First Hospital, Nanjing Medical University, No. 68 Changle Road, Nanjing, 210006 Jiangsu China; 2 State Key Laboratory of Bioelectronics, School of Biological Science and Medical Engineering, Southeast University, Si Pai Lou 2, Nanjing, 210096 China

**Keywords:** Urinary tract obstruction, Mesenchymal stem cells

## Abstract

Urethral stricture (US) remains a challenging disease without effective treatment options due to the high recurrence rate. This study aims to evaluate the preventive effect of uncultured adipose derived stromal vascular fraction (SVF) on urethral fibrosis in a rat model of US. Results demonstrated that US rats displayed hyperechogenic urethral wall with a narrowed lumen compared with sham rats, while SVF rats exhibited less extensive urethral changes. By histology, US rats showed obvious submucosal fibrosis in the urethral specimens, while SVF rats exhibited mild submucosal fibrosis with less extensive tissue changes. Furthermore, US rats showed increased gene and protein expression of collagen I (2.0 ± 0.2, 2.2 ± 0.2, all were normalized against GAPDH, including the following), collagen III (2.5 ± 0.3, 1.2 ± 0.1), and TGFβ1R (2.8 ± 0.3, 1.9 ± 0.2), while SVF cells administration contributed to decreased gene and protein expression of collagen I (1.6 ± 0.2, 1.6 ± 0.2), collagen III (1.8 ± 0.4, 0.9 ± 0.1), and TGFβ1R (1.8 ± 0.3, 1.3 ± 0.2), in parallel with the improvement of vascularization and increased expression of VEGF (1.7 ± 0.1) and bFGF (3.1 ± 0.3). Additionally, SVF served anti-inflammatory effect through regulation of inflammatory cytokines and cells, accompanied with conversion of the macrophage phenotype. Our findings suggested that uncultured SVF presented an inhibitory effect on stricture formation at an early stage of urethral fibrosis.

## Introduction

Urethral stricture (US) is a common disease with a high incidence in male patients, which causes serious impact on the life quality of patients and heavy healthcare costs^1^. Patients with US experience a decreased flow rate, which is accompanied by the potential complications including acute urinary retention, bladder and renal injury, and eventually renal failure. Current treatment options for US disease, including urethral dilatation and internal urethrotomy, are characterized by a high recurrence rate^2^. Open urethroplasty is taken for a valid and successful treatment, which is still associated with an overall recurrence rate of 15.6%, as well as various drawbacks such as invasive procedure, possible complications, and the need for special training and expertise^3^.

Though the exact mechanism remains unknown, urethral spongiofibrosis caused by excessive collagen production after infection or injury is the main pathology of US^4^. To improve treatment outcomes, anti-fibrotic drugs, for example halofuginone, mitomycoin C, etc., have been tested to limit the recurrence of US. However, there wasn’t sufficient therapeutic benefit observed after the administration of these medicines^5^. The intrinsic capacities of stem cells make them promising candidates for US treatment. Recently, adipose tissue-derived stem cells (ADSCs) have been explored to be capable of reducing urethral spongiofibrosis, enhancing tissue repair, and ultimately promoting functional recovery^6^. Previously, we showed that adipose-derived stromal vascular fraction (SVF) contained stem/progenitor cells, such as ADSCs, with excellent regenerative potential^7,8^. Administration of freshly isolated SVF cells was reported to be able to reduce renal and penile fibrosis^8,9^. SVF can be prepared simply and quickly without cell culture, and can be utilized with promising results in accelerating the repair of injured tissue. Thus, uncultured SVF cells have been suggested as an easy and safe candidate for alleviating tissue injury and promoting functional recovery in various tissues and organs^10^.

However, no attempt has so far been made to investigate whether uncultured SVF cells could attenuate urethral spongiofibrosis and prevent US formation. In this work, we aimed to explore the potential effect of uncultured SVF cells in alleviating urethral spongiofibrosis and promoting urethral tissue repair in a rat model of US.

## Methods

### Animals

Thirty male Sprague–Dawley rats (10 weeks old, 220–250 g) were used in this study. Rats were housed in a standard room with a 12 h light/dark cycle and controlled temperature and humidity. Animals were also provided with food and water ad libitum. All experiments were performed in accordance with the institutional and national guidelines for laboratory animals, and approved by the Ethics Committee of the Nanjing First Hospital, Nanjing Medical University. Also, the study was performed in compliance with the ARRIVE guidelines (http://www.nc3rs.org.uk/page.asp?id=1357).

### Study design

The rats were randomly divided into three groups: sham-operated group (Sham, n = 10); US + phosphate-buffered saline (PBS) group (US, n = 10), and US + SVF group (SVF, n = 10). In the sham group, rats received 100 μl saline injections to the urethral wall without incision of the urethra. In the other two groups, human recombinant transforming growth factor beta 1 (TGF-β1, 1 μg in 100 μl saline, PeproTech, USA) was injected to the urethral wall to induce US formation. On the following day, SVF cells were isolated and suspended in 100 μl PBS at a dosage of 2 × 10^6^ cells and transplanted into the urethral wall through single local injection in the SVF group, while 100 μl PBS was injected instead in the US group. Ultrasound examination was performed on the rats of each group four weeks later, after which the animals were euthanized. Then, urethral tissues were harvested and used for the subsequent experiments. All surgical procedures were conducted by sterile techniques under anesthesia with intraperitoneally injected 100 mg/kg ketamine.

### Isolation and characterization of SVF

According to our previously published protocol, autologous SVF cells were isolated from epididymal adipose tissue^7,11^. Briefly, after been washed thrice with ice-cold sterile PBS, adipose tissue was cut into small pieces and digested with 0.075% type I collagenase by gently shaking for 30–40 min at 37 °C. After centrifugation and repetitive wash steps, SVF cell pellet was treated with Red Blood Cell Lysis Buffer, resuspended with PBS, and then counted with an automated cell counter.

Flow cytometric analysis was performed to detect the cell markers of uncultured SVF cells with the following antibodies: phycoerythrin (PE) conjugated anti-CD11b/c (BioLegend, San Diego, CA, USA), PE conjugated anti-CD34 (Bioss Antibodies Inc., Woburn, MA, USA), PE conjugated anti-CD106 (Bioss Antibodies Inc.), PE conjugated anti-CD133 (Novus Biologicals, USA), fluorescein isothiocyanate (FITC) conjugated anti-CD31 (Bioss Antibodies Inc.), FITC conjugated anti-CD90 (Bioss Antibodies Inc.), FITC conjugated anti-vascular endothelial growth factor receptor-2 (VEGFR-2) (Bioss Antibodies Inc.), and FITC conjugated anti-CD45 (BioLegend). The labeled SVF cells were analyzed by using FACSCaliber (BD Biosciences, San Diego, CA). A negative control was set by using an isotype-matched IgG for each antibody.

### US model

US model was created as previously described^6^. After each rat was anesthetized with ketamine (100 mg/kg), a PE-90 urethral catheter was gently inserted into the urethra. An incision of the ventral penile skin was made to expose the urethra. Then, each rat underwent a single injection of 1 μg human recombinant TGF-β1 in 100 μl of saline to the urethral wall with a 30G needle. Five minutes after the injection, four partial incisions of the penile urethra were conducted, with a 23G needle, through all layers of the urethral wall to ensure that the catheter could be visualized. At last, the urethral catheter was removed, and the penile skin was sutured with 5–0 absorbable sutures.

### Microultrasound assessment

At four weeks following the injury, rats were received microultrasound assessment with the Vevo 2100 system (Visual-Sonics Inc., Toronto, Canada) to evaluate the signs of fibrosis in the urethra. The procedure was conducted in a blinded manner and repeated three times.

### Histopathology and immunofluorescent staining

The urethral tissues were fixed, dehydrated, embedded in paraformaldehyde, and then sectioned at 5-μm for hematoxylin and eosin (H&E) and Masson’s trichrome staining. Slides were detected by a pathologist who was blinded to the groups by a standard light microscopy (Olympus BX53, Tokyo, Japan). Angiogenesis of urethral tissues, infiltration of inflammatory cells (macrophages, neutrophils and T cells), as well as expression of inflammatory cytokines were analyzed by immunofluorescent staining. The antibodies used for immunofluorescent staining are list as following: anti-CD31 and anti-CD34 (Abcam, Cambridge, MA, USA) for angiogenesis, anti-CD68 for pan macrophages, anti-iNOS for M1 macrophages, anti-CD163 for M2 macrophages, anti-Ly6G for neutrophils, anti-CD3 for T cells, anti-TNF-α and anti-IL-10 for inflammatory cytokines. Immunofluorescent staining was carried out according to previous published protocol^12^. In brief, the sections were blocked, incubated with primary antibodies at 4 °C, followed by being stained with IgG Alexa Fluor 633 or IgG Alexa Fluor 488. At last, the cellular nucleus was stained with DAPI.

### Gene expression

The total urethral tissue RNA was extracted by using Trizol reagent (Invitrogen, Carlsbad, CA) and converted into cDNA with Prime Script® RT Master Mix (TaKaRa, Japan) according to the manufacturer’s instructions. Real-time PCR was performed in triplicate with the SYBR® Premix Ex Taq™ (TaKaRa) on the Applied Biosystems 7500 real-time PCR system. The relative expression of each target gene was normalized to the internal glyceraldehyde-3-phosphate dehydrogenase (GAPDH). The primer sequences were designed according to the data from GenBank and evaluated by nucleotide blaststandard search to avoid any cross-reactivity with other known sequences (Table 1).

**Table 1:** Description of the primer sequences.

Primer	Sequence (5′–3′)	Amplicon size (bp)
**Collagen I**		184
Forward	GGAGAGAGCATGACCGATGG	
Reverse	GGGACTTCTTGAGGTTGCCA	
**Collagen III**		161
Forward	CCCTGAACTCAAGAGCGGAG	
Reverse	TCTCAGCACCAGCATCTGTC	
**TGF-β1R**		154
Forward	CTGCCTGCTTCTCATCGTGT	
Reverse	GGCTCTGGT GTCTGTTTCAA	
**GAPDH**		103
Forward	GGCCTTCCGTGTTCCTACC	
Reverse	CGCCTGCTTCACCACCTTC	

### Western blot analysis

Total protein was extracted from retrieved urethral tissues with RIPA lysis buffer including a cocktail of protease inhibitor (Roche, Mannheim, Germany). The protein concentration was assessed with a BCA protein assay kit (Beyotime, Shanghai, China). The expression of target proteins was determined by western blot analysis according to our previously published protocol^8^. Primary antibodies against Collagen I (Abcam), Collagen III (Abcam), TGF-β1R (Abcam), vascular endothelial growth factor (VEGF, Santa Cruz Biotechnology Inc., Santa Cruz, CA, USA), and basic fibroblast growth factor (bFGF, Bioss Inc.) were used in this study. Anti-GAPDH antibody was used as an internal standard. The density of immunoreactive proteins was determined by using the Gel-Pro32 software (Media Cybernetics). The original blot of each antibody can be found in Supplementary information.

### Statistical analysis

All data were shown as means ± standard deviation. Statistical analysis for multiple comparisons among all groups was conducted with a one-way analysis of variance (ANOVA) followed by the post hoc Tukey test. *p* < 0.05 was considered statistically significant.

### Ethical statement

All applicable international, national, and/or institutional guidelines for the care and use of animals were followed. All experimental protocols were approved by the Ethics Committee of the Nanjing First Hospital, Nanjing Medical University.

## Results

### Characterization of SVF

**Figure 1:**
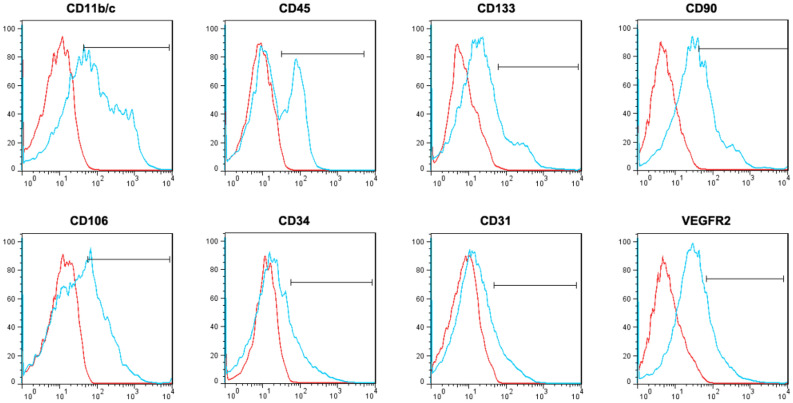
Representative flow cytometry histograms of uncultured adipose derived stromal vascular fraction (SVF). Uncultured SVF cells expressed hematopoietic (CD11b/c, CD34, CD45, and CD133), mesenchymal (CD90 and CD106), and endothelial (CD31 and VEGFR2) markers. The red lines represent isotype controls.

Flow cytometric analysis demonstrated that uncultured SVF cells expressed hematopoietic (CD11b/c [39.35 ± 7.62%], CD34 [15.50 ± 5.23%], CD45 [40.50 ± 7.22%], and CD133 [15.43 ± 4.57]), mesenchymal (CD90 [38.60 ± 6.11%] and CD106 [32.12 ± 9.21%]), and endothelial (CD31 [13.32 ± 5.50%] and VEGFR2 [20.37 ± 5.89%]) markers, indicating a heterogeneous population of SVF cells (Fig. 1).

### Microultrasound

**Figure 2:**
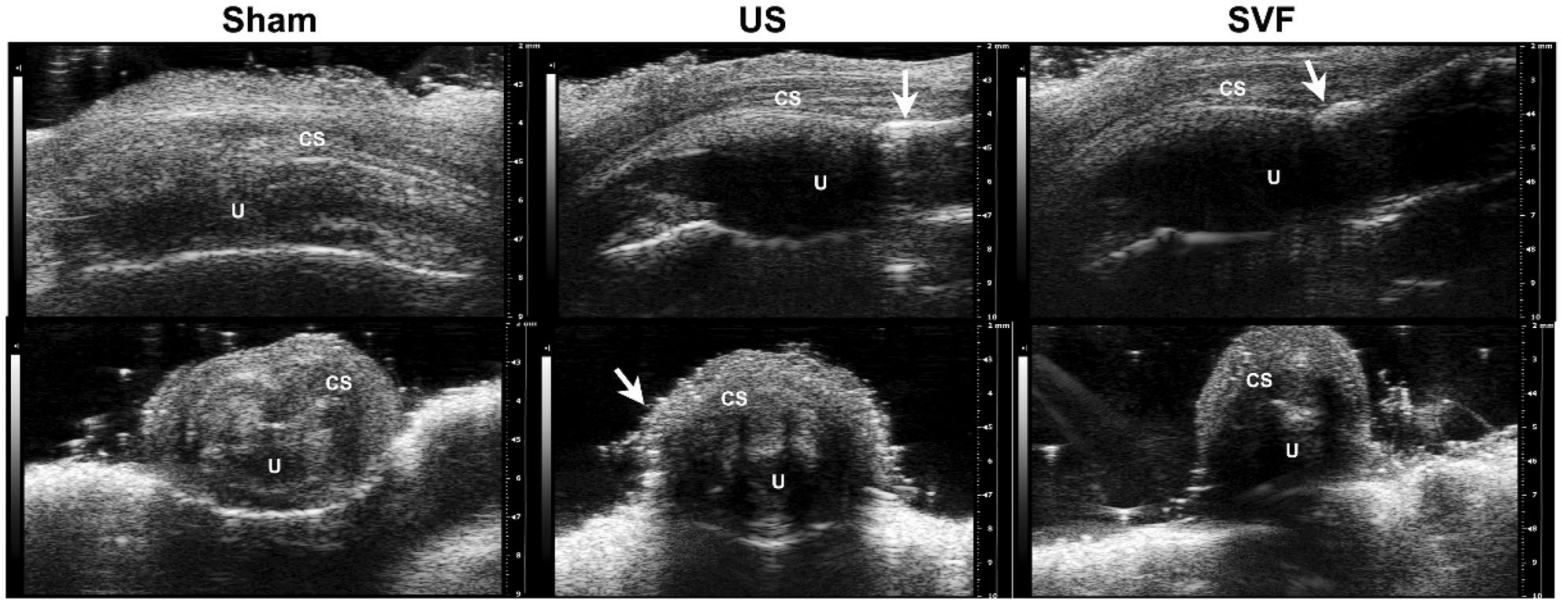
Representative microultrasound images of penile urethras in sham, US and SVF groups at 4 weeks after surgery. White arrows indicate sites of hyperechogenic tissues and narrowing of the urethral lumen in the US and SVF groups. *CS* corpus spongiosun, *U* urethral lumen.

Microultrasound analysis showed hyperechogenic urethral walls and narrowing of the urethral lumen in both US and SVF groups compared with that in sham rats. Whereas, less extensive areas of hyperechogenic tissue and urethral stricture could be found in the SVF treated rats (Fig. 2).

### Uncultured SVF alleviates urethral fibrosis

**Figure 3:**
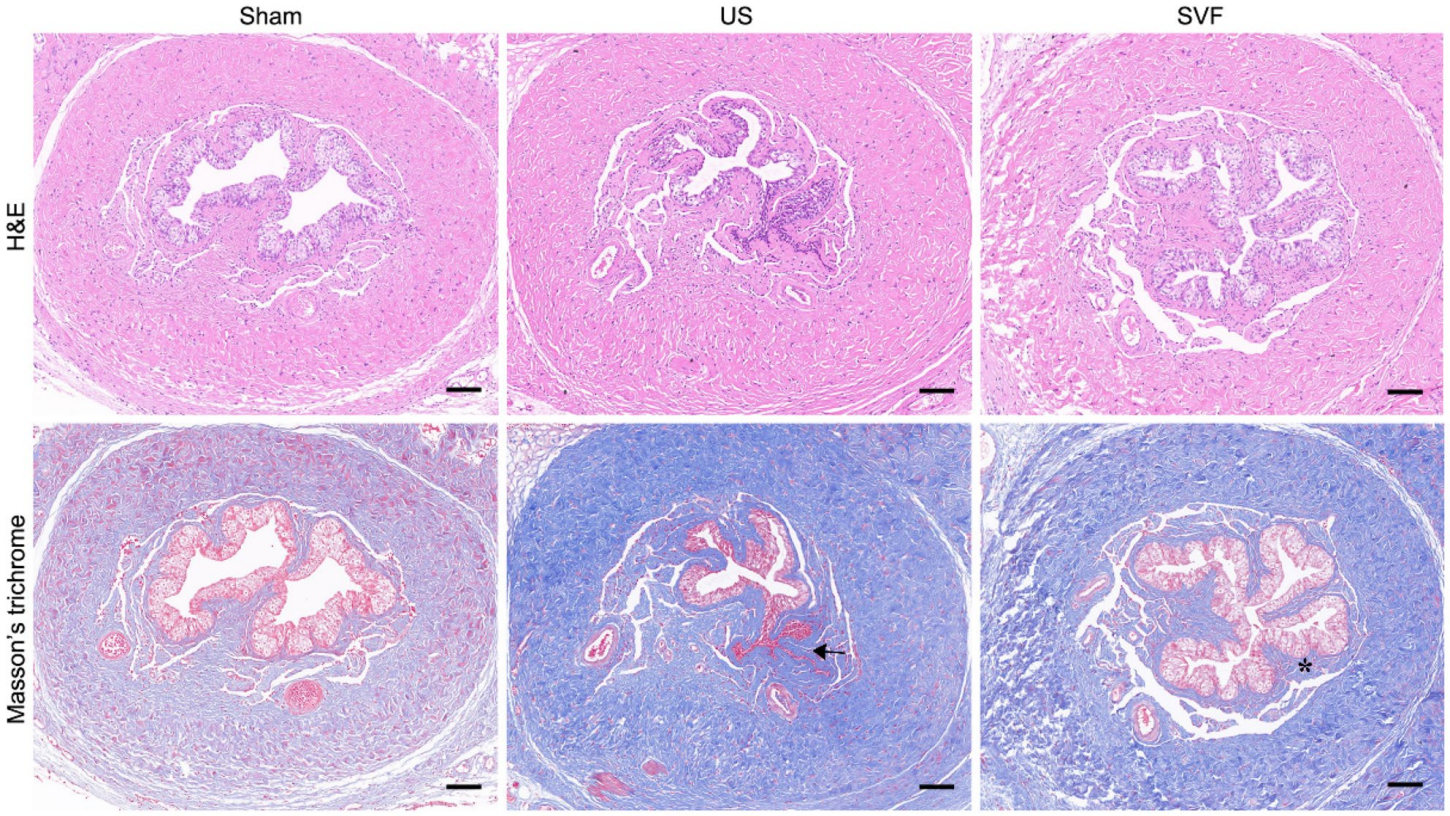
Representative microscopic findings of hematoxylin and eosin (H&E) and Masson’s trichrome staining in rat urethral tissues in sham, US and SVF groups at 4 weeks after surgery. Scale bar = 100 μm. US rats showed densely packed collagen bundles and sparse smooth muscle in the urethral tissues. Black arrows indicate the sites of collagen deposition with few vascular. SVF rats exhibited mild submucosal fibrosis with less extensive tissue changes. Asterisk indicate the sites of less collagen deposition with visible vascular.

Microscopic findings of representative H&E and Masson’s trichrome staining showed obvious submucosal fibrosis with densely packed collagen bundles and sparse smooth muscle in the urethral specimens of US rats. However, spongious urethra from SVF treated rats exhibited mild submucosal fibrosis with less extensive tissue changes (Fig. 3).

### Uncultured SVF reduces collagen expression in rats with urethral stricture

**Figure 4:**
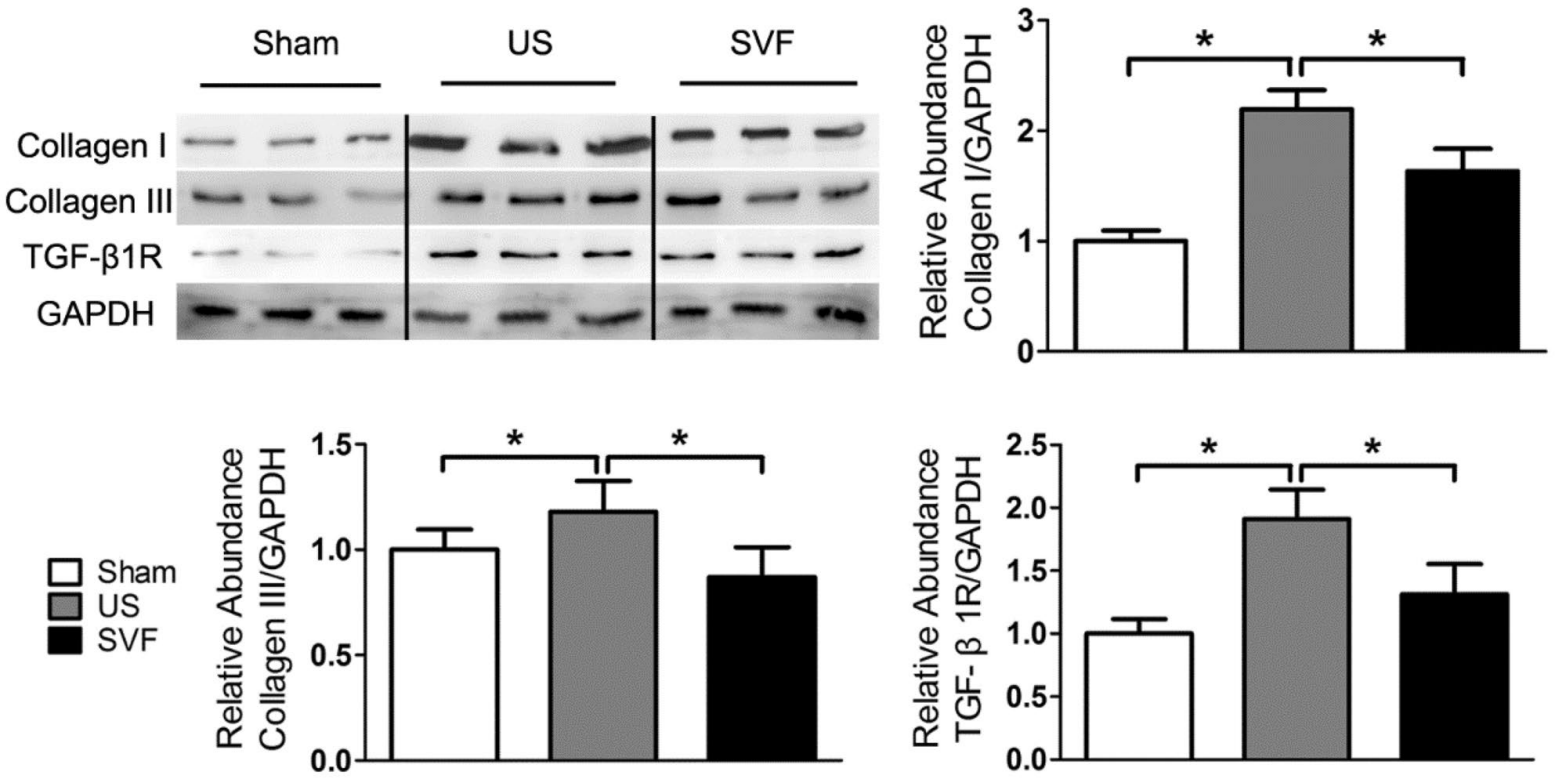
Protein expression of Collagen I, Collagen III and TGFβ1R in urethral tissues were measured by western blot analysis in sham, US, and SVF groups at 4 weeks after surgery. Western blots were quantified with data expressed as relative abundance of Collagen I/GAPDH, Collagen III/GAPDH, and TGFβ1R/GAPDH. Asterisk indicates *p* < 0.05, which was considered significant differences in protein expression.

**Figure 5:**
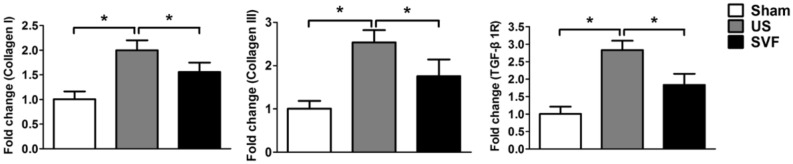
Gene expression of collagen I, collagen III, and TGFβ1R in urethral tissues were measured by real-time PCR in sham, US, and SVF groups at 4 weeks after surgery. Asterisk indicates *p* < 0.05, which was considered significant differences in gene expression.

Collagen expression was evaluated by western blot to confirm urethral fibrosis. Compared with sham rats, urethral specimens in US rats showed significantly increased expression of collagen I, collagen III, and TGFβ1R. Administration of SVF resulted to a significant reduction of collagen I, collagen III, and TGFβ1R in US rats (Fig. 4). Moreover, gene expression of collagen I, collagen III, and TGFβ1R was determined by real-time PCR. The results demonstrated that high levels of gene expression for collagen I, collagen III, and TGFβ1R were detected in the urethral tissues of US rats, compare with that in sham rats. Whereas, the increase of collagen I, collagen III, and TGFβ1R expression was significantly inhibited by the administration of SVF (Fig. 5).

### Uncultured SVF promotes vascularization in rats with urethral stricture

**Figure 6:**
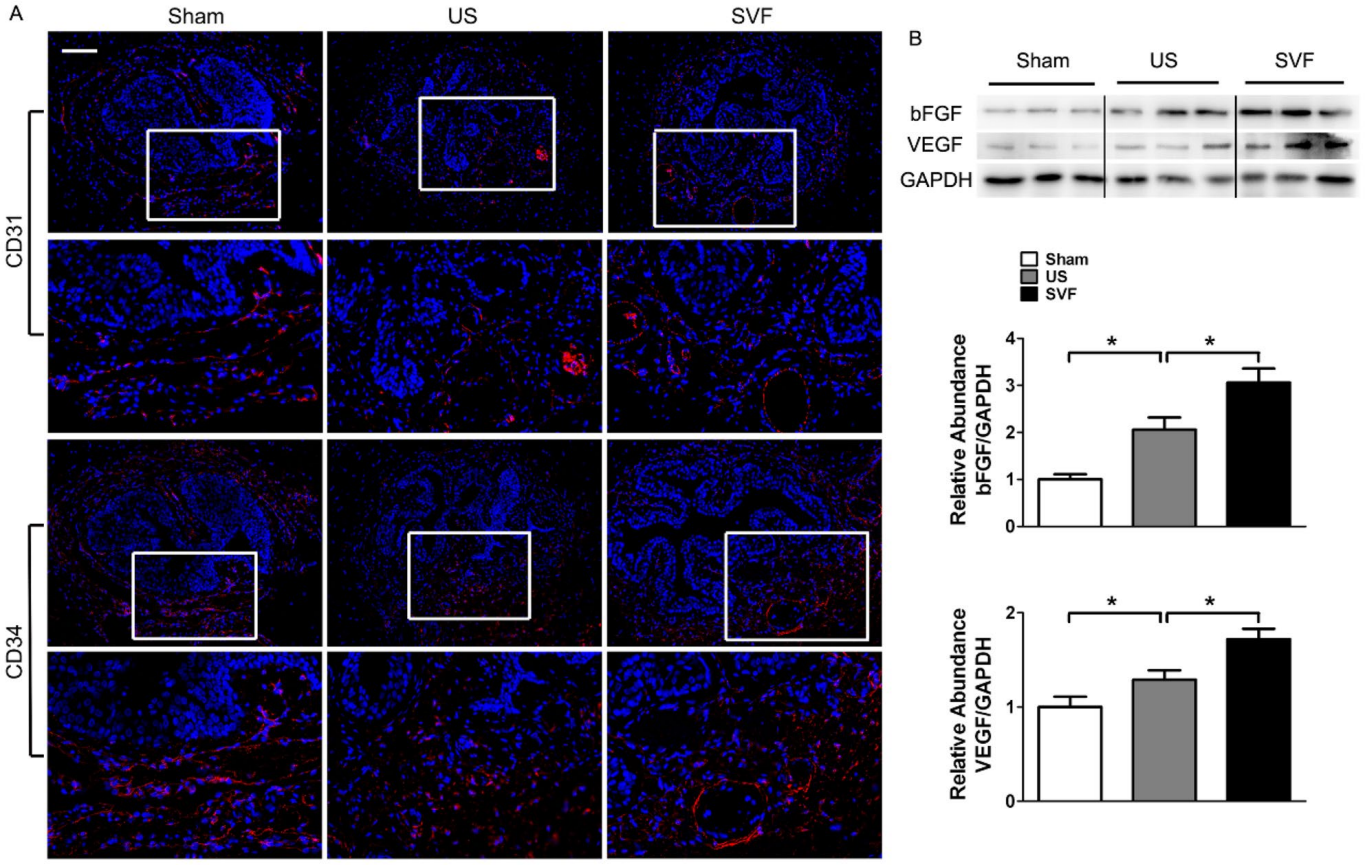
The angiogenic effect of SVF in urethral tissues. (**A**) CD31 and CD34 expression was evaluated by immunofluorescent staining of urethral tissues in sham, US, and SVF groups at 4 weeks after surgery. Scale bar = 100 μm. (**B**) Expression of VEGF and bFGF in urethral tissues were measured by western blot analysis. Western blots were quantified with data expressed as relative abundance of VEGF/GAPDH, and bFGF/GAPDH. Asterisk indicates *p* < 0.05.

Immunofluorescent staining revealed significantly reduced expression of CD31 and CD34 in the urethral tissues of US rats. Expression of CD31 and CD34 was enhanced by the treatment of SVF, indicating the improvement of vascularization in urethral tissues with stricture. Meanwhile, angiogenesis-related proteins, including VEGF and bFGF, were evaluated by western blot. Increased levels of both VEGF and bFGF were detected in SVF treated animals, which were higher than that in US group (Fig. 6).

### Uncultured SVF performed anti-inflammatory effect in rats with urethral stricture

**Figure 7:**
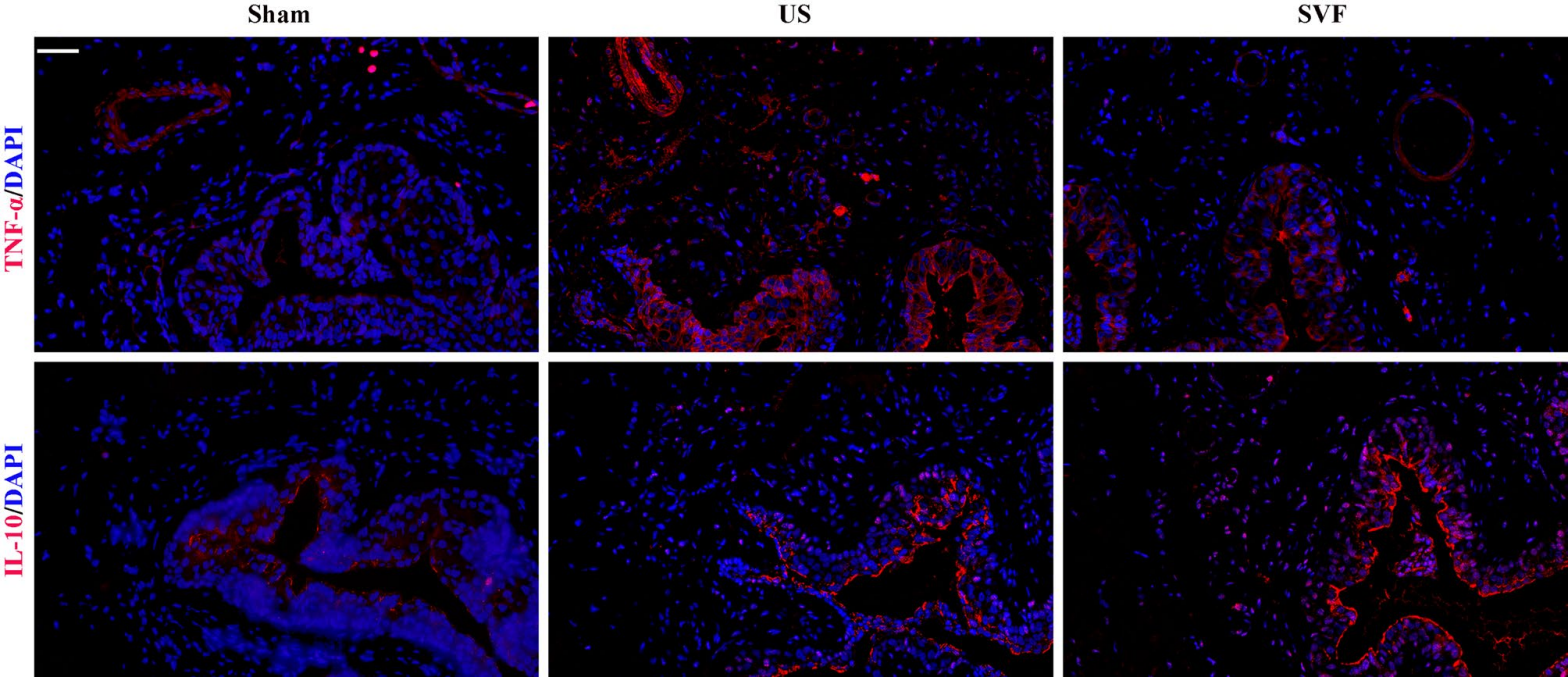
Expression of inflammatory cytokines (TNF-α and IL-10) in the urethral tissues was evaluated by immunofluorescent staining in sham, US, and SVF groups at 4 weeks after surgery. Scale bar = 50 μm.

**Figure 8:**
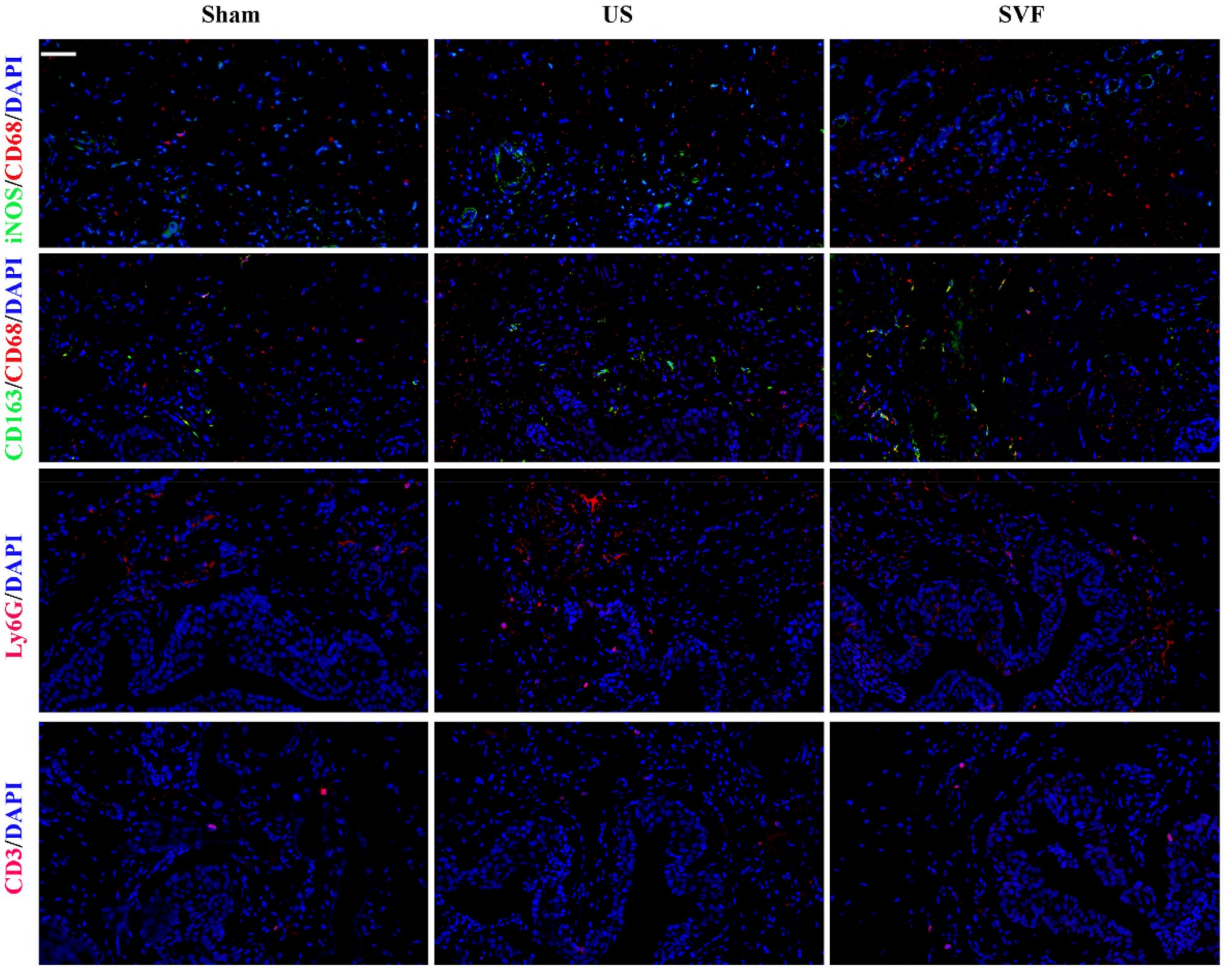
The infiltration of inflammatory cells in urethral tissues was evaluated by immunofluorescent staining (anti-CD68 for pan macrophages, anti-iNOS for M1 macrophages, anti-CD163 for M2 macrophages, anti-Ly6G for neutrophils, anti-CD3 for T cells) in sham, US, and SVF groups at 4 weeks after surgery. Scale bar = 50 μm.

Immunofluorescent staining revealed significantly increased expression of TNF-α and IL-10 in the urethral tissues of US rats. Reduced expression of TNF-α as well as increased expression of IL-10 were detected in SVF treated rats, indicating the anti-inflammatory effect of SVF in urethral tissues with stricture (Fig. 7). Meanwhile, infiltration of M1 macrophages (iNOS+/CD68+) and M2 macrophages (CD163+/CD68+) were demonstrated in the urethral tissues of US rats. However, significantly increased infiltration of M2 macrophages was detected in SVF group. In addition, elevated expression of Ly6G was detected in US rats, which indicated the infiltration of neutrophils, while SVF treatment could reduce their infiltration. However, there was no significant difference in CD3 expression among the three groups (Fig. 8).

## Discussion

Currently, US remains a challenging disease without effective treatment options over the last few decades, due to the high recurrence rate. Various iatrogenic and non-iatrogenic factors, including external trauma, urethritis, urethral catheterization, endoscopic procedures, etc., may eventually cause US development^13,14^. Therefore, efficiently preventing the formation of urethral fibrosis when the above etiology occurs might be an effective method to treat US. This study firstly demonstrated that administration of uncultured SVF cells after urethral injury could attenuate urethral fibrosis and therefore inhibit the development of US in a rat model. Nonexpanded SVF cells have gained wide attention with extensive translational significance in cell-based therapy for tissue repair and regeneration, and demonstrated safety and efficacy in various clinical practice^15–18^. Therefore, transplantation of nonexpanded SVF cells may provide a novel therapeutic approach to alleviate the formation of urethral fibrosis, which highlights the potential clinical implications for the treatment of US (Supplementary information).

To generate a reproducible animal model of US, TGF-β1 was introduced after traumatic urethral injury by a needle. TGF-β1 is a profibrotic factor that can induce the synthesis of extracellular matrix proteins secreted by fibrogenic cells, thereby playing an important role in orchestrating fibrosis^19,20^. Researchers demonstrated an increased expression level of TGF-β1 receptor in human urethral stricture specimens, and proposed a simple and reproducible technique to create urethral fibrosis by TGF-β1 injection in a rat model^21^. Through a duplicated TGF-β1 injection technique, another study was conducted to ensure reproducible urethral spongiofibrosis in a urethral injury model. Stricture formation was further confirmed by in vivo microultrasound measurement that revealed the narrowing of the urethral lumen^6^. Our results verified the reproducible technique for inducing stricture formation by injection of TGF-β1 after urethral injury.

Deposition of extracellular matrix components is a characteristic feature of urethral fibrosis, including the upregulation of collagen I and collagen III^21,22^. Studies showed that both allogeneic and xenogenic ADSCs injection could decrease TGF-β1 induced urethral fibrosis with the downregulation of collagen I and collagen III^6,23^. Adipose tissue derived SVF, consisting of heterogeneous cell populations including ADSCs, is reported to be capable of preventing fibrotic changes of penile fibrosis in acute and chronic phase of Peyronie’s disease^9,24^. We previously found that local injection of SVF cells contributed to the attenuation of ischemia–reperfusion injury induced renal fibrosis^8^. In the present study, administration of SVF cells prevented urethral fibrosis as seen by a list of outcome measurements, including microultrasound, histology, gene and protein expression analyses. The upregulation of collagen I and collagen III in US tissues was attenuated by SVF injection in both gene and protein levels.

As a profibrotic factor, TGF-β1 promotes the process of fibrosis through binding to its receptor. Increased expression of TGF-β1 receptor was demonstrated in the human and rat urethras with US^21^. In our study, high levels of gene and protein expression for TGF-β1 receptor were detected in US rats, and decreased by the injection of SVF, which may partly account for the preventing effect of SVF on fibrosis. In addition, the improvement of vascularization in urethral stricture might be another contributor for the attenuation of fibrosis by SVF cells, due to their secretion of pro-angiogenic factors, including VEGF, bFGF, and so on. Urethra is reported to be sensitive to ischemia that can induce fibrosis in the injured tissues^25,26^. SVF has been verified to promote angiogenesis by secreting pro-angiogenic factors^10^. Furthermore, inflammatory cells and cytokines are considered to play key roles in fibrosis. Previous studies revealed that SVF could present anti-inflammatory properties after being transplanted in vivo^27,28^. In our study, SVF administration result in reduced expression of pro-inflammatory cytokine (TNF-α), increased expression of anti-inflammatory cytokine (IL-10), accompanied with reduced infiltration of neutrophils, indicating that SVF might serve anti-inflammatory effect during the urethral fibrosis. Additionally, more increased infiltration of M2 macrophages of urethral tissues was detected in SVF treated rats compared with US rats injected with PBS, suggesting SVF transplantation contributed to the conversion of the macrophage phenotype, which is confirmed by previous study^28^. Adipose tissues derived mesenchymal stromal cells induced macrophage polarization led to a pro-repair phenotype in injured tissue^29^. However, a better understanding of the potential mechanisms involved in the inhibiting effect of SVF on urethral fibrosis needs further investigation in the future study.

Even though nonexpanded SVF was found to attenuate urethral fibrosis through a paracrine manner, whether local injected SVF cells could survival or differentiate into target cells was still unclear. It may be beneficial for such issue by using cell tracing technique to imaging transplanted cells with fluorescent dye^12,30^. Furthermore, SVF cells were administrated in the early phase of urethral injury to prevent the process of fibrosis. However, in some real world situations, SVF injection may not occur in unison with urethral injury. Urethral fibrosis may have been established when the patients received SVF injection. Further study is needed to explore whether SVF treatment is able to reduce established urethral fibrosis. At last, due to the high recurrence rate of current treatment for US disease, such as urethroplasty, further research is needed to investigate whether SVF injection can be conducted simultaneously during the process of urethroplasty to prevent fibrosis and reduce the recurrence rate of US.

## Conclusions

The results of our study revealed that uncultured SVF cells presented an inhibitory effect on stricture formation in a rat model of urethral fibrosis. This study may provide a therapeutic option to attenuate the formation of urethral fibrosis, and suggest the potential clinical implications for the treatment of US.

## Supplementary Information

Supplementary Information.
