# Supplementary material for: Effect of uncultured adipose-derived stromal vascular fraction on preventing urethral stricture formation in rats
Source: Sci Rep. 2022 Mar 4;12:3573. doi: 10.1038/s41598-022-07472-1 (PMC8897427; doi:10.1038/s41598-022-07472-1)
Supplement: Supplementary file 1 — Supplementary Information. [file 41598_2022_7472_MOESM1_ESM.docx]

**Effect of Uncultured Adipose-derived Stromal Vascular Fraction on Preventing Urethral Stricture Formation in Rats**

Liuhua Zhou^1^, Tianli Yang^1^, Feng Zhao^1^, Kaiwei Song^1^, Luwei Xu^1^, Zhongle Xu^1^, Changcheng Zhou^1^, Zhiqiang Qin^1^, Zheng Xu^1^, Ran Wu^1^, Hua Xu^2,^*, Ruipeng Jia^1,^*

^1^Department of Urology, Nanjing First Hospital, Nanjing Medical University, No.68 Changle Road, Nanjing, Jiangsu 210006, China.

^2^State Key Laboratory of Bioelectronics, School of Biological Science and Medical Engineering, Southeast University, Si Pai Lou 2, Nanjing 210096, China.

Fig.4 Collagen I





This is the full-length gel and blot of Collagen I in Fig.4. From the right side, the first three blots represent the expression level of Collagen I in the urethral tissues of the SVF group. The second and third three blots represent the expression level of Collagen I in the urethral tissues of the US group and Sham group, respectively.

Fig.4 Collagen III





This is the full-length gel and blot of Collagen III in Fig.4. From the right side, the first three blots represent the expression level of Collagen III in the urethral tissues of the SVF group. The second and third three blots represent the expression level of Collagen III in the urethral tissues of the US group and Sham group, respectively.

Fig.4 TGF-β1R





This is the full-length gel and blot of TGF-β1R in Fig.4. From the right side, the first three blots represent the expression level of TGF-β1R in the urethral tissues of the SVF group. The second and third three blots represent the expression level of TGF-β1R in the urethral tissues of the US group and Sham group, respectively.

Fig4. GAPDH





This is the full-length gel and blot of GAPDH in Fig.4. From the right side, the first three blots represent the expression level of GAPDH in the urethral tissues of the SVF group. The second and third three blots represent the expression level of GAPDH in the urethral tissues of the US group and Sham group, respectively.

Fig.6 bFGF





This is the full-length gel and blot of bFGF in Fig.6. From the right side, the first three blots represent the expression level of bFGF in the urethral tissues of the SVF group. The second and third three blots represent the expression level of bFGF in the urethral tissues of the US group and Sham group, respectively.

Fig.6 VEGF





This is the full-length gel and blot of VEGF in Fig.6. From the right side, the first three blots in the second row represent the expression level of VEGF in the urethral tissues of the SVF group. The second and third three blots in the second row represent the expression level of VEGF in the urethral tissues of the US group and Sham group, respectively.

Fig.6 GAPDH





This is the full-length gel and blot of GAPDH in Fig.6. From the right side, the first three blots represent the expression level of GAPDH in the urethral tissues of the SVF group. The second and third three blots represent the expression level of GAPDH in the urethral tissues of the US group and Sham group, respectively.
